# Nurse and Patient Outcomes in Private and Public Hospitals in South Africa During the COVID‐19 Pandemic: A Cross‐Sectional Study

**DOI:** 10.1155/jonm/1853384

**Published:** 2026-05-03

**Authors:** Siedine K. Coetzee, Linda H. Aiken, Alwiena J. Blignaut, Anelle Erasmus, Nicholin Scheepers, Babalwa Tau, Nokwanda Edith Bam, Erika Fourie

**Affiliations:** ^1^ NuMIQ Research Focus Area, North-West University, Potchefstroom, 2520, South Africa, nwu.ac.za; ^2^ Center for Health Outcomes and Policy Research, University of Pennsylvania School of Nursing, Philadelphia, Pennsylvania, USA; ^3^ School of Mathematical and Statistical Sciences, Pure and Applied Analytics, North-West University, Potchefstroom, South Africa, nwu.ac.za

**Keywords:** hospitals, nurse outcomes, patient outcomes, practice environment, South Africa, survey

## Abstract

**Background:**

Nurse and patient outcomes in South Africa were poor before COVID‐19 and are believed to have worsened during and after the pandemic. Limited evidence exists on modifiable organisational factors contributing to these outcomes hindering targeted interventions.

**Purpose:**

This paper aims to develop a better understanding of potentially modifiable organisational factors of hospitals that, if addressed, would likely contribute to improving nurse wellbeing and retention, and quality and safety of patient care.

**Methods:**

Data were collected from 143 private and public hospitals (*n* = 4298 nurses) across South Africa using a cross‐sectional survey. Independent variables included working time with COVID‐19 patients, incidence of death and dying, resources, staffing, and the practice environment; dependent variables focused on nurse outcomes (job satisfaction, intent to leave, burnout, mental and physical health) and patient outcomes (quality of care and patient safety).

**Results:**

Nurse and patient outcomes were worse in public compared to private hospitals. Favourable practice environments had the strongest association with nurse and patient outcomes, followed by staffing and resources. Within the practice environment, nurse management, leadership and support of nurses showed the greatest association with job satisfaction (OR = 4.71^∗∗^; 95% CI = 3.97–5.58), lower intent to leave (OR = 2.81^∗∗^; 95% CI = 2.33–3.38) and more favourable mental health (OR = 2.58^∗∗^; 95% CI = 2.19–3.04). Greater nurse participation in hospital affairs was associated with more favourable nurse assessments of quality of care (OR = 3.74^∗∗^; 95% CI = 3.22–4.33 to OR = 6.51^∗∗^; 95% CI = 3.81–4.95) and patient safety (OR = 4.35^∗∗^; 95% CI = 3.81–4.95).

**Conclusion:**

Interventions to improve nurse wellbeing and retention as well as quality and safety of care should focus on improving hospital practice environments, specifically nurse manager expertise, nurse leadership, nurse participation in hospital affairs, and adequate staffing and resources.

**Highlights**
• What is Already Known?
◦ Hospitals in developing countries such as South Africa have long had poor nurse wellbeing and retention, and unfavourable patient outcomes even prior to the COVID‐19 pandemic.
◦ Evidence is lacking on actionable strategies to improve nurse wellbeing, retention, patient safety, and quality of patient care.
• What This Paper Adds?
◦ This study identified modifiable organisational factors in hospitals that, if successfully targeted, hold potential for improving nurse wellbeing and retention as well as patient safety and quality of care in hospitals in South Africa and beyond.
◦ Of all retention interventions listed postpandemic, the focus in South Africa should be directed towards improving hospital practice environments, specifically nurse manager expertise, leadership development, greater nurse participation in hospital affairs and adequate nurse staffing and resources.

## 1. Introduction

South Africa (SA) has a dual public and private healthcare system serving a diverse and divided population. Despite significant progress towards Universal Health Coverage, the system faces several challenges [1]. Among the main challenges is a shortage of health professionals and clinicians, made worse by poor retention [1].

The number one priority and embodiment of any health system is human resources, especially physicians and nurses [2]. Nurses make up 56% of healthcare workers in SA [2] and 59% of human resources for health in the world [3]. Thus, nurses play a pivotal role in achieving equitable access to care and the Sustainable Development Goals [2, 3]. Before the COVID‐19 pandemic there was a global shortfall of approximately 5.7 million nurses [3], with approximately 89% of the shortage concentrated in low‐ and lower‐middle‐income countries [4]. In SA the nurse shortage was estimated at 26,000–62,000, mostly ascribed to escalating healthcare needs of a growing population, the quadruple burden of disease, retirement of experienced nurses, unfavourable working conditions, and poor salaries [5]. Postpandemic, this shortfall has increased to 7 million nurses globally [3] and 260,458 nurses in SA [6].

The pandemic inflicted unprecedented damage on the health system, nurse outcomes and patient outcomes [4]. Developing countries such as SA had been deeply affected by poor nurse and patient outcomes prior to the pandemic [1], with sub‐Saharan Africa having the highest levels of poor nurse outcomes [7]. Globally, these trends increased during the pandemic, with poorer nurse outcomes including physical health symptoms [8], mental health symptoms [9], burnout [10], lower job satisfaction and intention to leave [11]. Nurses also reported poorer patient outcomes, including decreased quality of care [11–13], compromised patient safety [12] and a higher prevalence of adverse events during the pandemic [13].

Systematic reviews highlight the main organisational factors impacting nurse and patient outcomes during the pandemic as being longer working time in quarantine areas, working in a high‐risk environment, unfavourable practice environments, inadequate and insufficient supplies and human resources, decreased social support, increased workload, and limited information or specialised training regarding COVID‐19 [14, 15].

Globally, countries are developing urgent and co‐ordinated policy responses to try and increase nurse supply, recruitment, and retention [2, 4]. While SA has done much to focus on nurse supply and recruitment [2], there has been less focus on retention, specifically postpandemic. This paper aims to develop a better understanding of potentially modifiable organisational factors of hospitals that, if addressed, would likely contribute to improving nurse wellbeing and retention, and quality and safety of patient care.

## 2. Materials and Methods

This study incorporated a quantitative, cross‐sectional, analytical design of 143 public and private SA hospitals employing their nurses as informants (*n* = 4298) via surveys about working conditions and nurse and patient outcomes in those hospitals.

### 2.1. Sample and Setting

SA has a public healthcare sector serving 83.5% of the population, who have no health insurance, and a private healthcare sector. Twenty‐seven public hospitals out of a total of 287 public hospitals were included in the study sample. In each province, the largest central or tertiary hospital was purposively selected. The provincial and district hospitals closest to the selected central or tertiary hospital were then included to ensure representation across all levels of public hospitals. A purposive sampling approach was therefore used to achieve representation while also accommodating the extended ethical approval process during the COVID‐19 pandemic. The application was submitted to the National Health Research Database on 02/07/2020, and goodwill consent from the final hospital was obtained on 12/04/2022. SA has approximately 216 private hospitals, of which about 75%–80% are owned by four major hospital groups. All four of these private hospital groups were included in the study, representing 116 hospitals across SA. Specialist hospitals were excluded. Initially, ethical approval was sought only for the largest hospital from each hospital group in every province. However, the respective hospital ethics committees requested that all hospitals within the participating groups be included in the study and that the originally planned paper‐based surveys be replaced with online surveys. All inpatient units within sampled hospitals were selected, with a total‐population sample of all categories of nurses, including (registered nurses [4‐year degree or diploma], enrolled nurses [2‐year diploma] and auxiliary nurses [1 year certificate]) (*n* = 2213 from public hospitals and *n* = 2085 from private hospitals).

### 2.2. Data Collection

Nurses were surveyed between April 2021 and June 2022. In the private sector, data collection took place via online self‐report surveys, which was preferred by private hospital managers to limit the possibility of COVID‐19 exposure. The public sector hospitals preferred paper‐based surveys due to dissemination constraints. Both survey forms contained identical questions.

### 2.3. Nurse Outcomes

Job dissatisfaction and intention to leave their employer were measured by single‐item questions previously validated in multiple large, published studies [16]. Job satisfaction was measured using a 4‐point Likert scale dichotomised into “dissatisfied” and “satisfied”. Intention to leave was measured with a nominal scale of “yes” and “no” [16]. Burnout was measured using the 9‐item emotional exhaustion subscale of the Maslach Burnout Inventory [17]. Respondents were classified as having high levels of burnout if their score was ≥ 27 [17]. Physical and mental health were measured using the Global Health Short Form—a Patient‐Reported Outcomes Measurement Information System (PROMIS) instrument [18]. Respondents were classified as having poor mental health and physical health when total scores were higher than 55, respectively [18].

### 2.4. Patient Outcomes

When recommending the hospital to family and friends (4‐point scale: 1 = definitely yes, 4 = definitely no; responses 1‐2 were dichotomised as positive), readiness to manage care after discharge (4‐point scale: 1 = very confident, 4 = not at all confident; responses 1‐2 were dichotomised as positive), confidence that management will act to resolve problems in patient care (4‐point scale: 1 = very confident, 4 = not at all confident; responses 1‐2 were dichotomised as positive), and quality of nursing care (4‐point scale: 1 = excellent, 4 = poor; responses 1‐2 were dichotomised as positive), data were provided by nurses in response to single‐item questions that have been shown to be highly associated with mortality and other patient outcomes [19]. Overall safety grade (5‐point scale: 1 = excellent, 5 = failing; responses 1–3 were dichotomised as positive) data were provided by nurses [19]. Data were dichotomised using the same variables and cut‐off scores as other national [20] and international articles [16].

### 2.5. Organisational Factors

The practice environment was measured using the validated Practice Environment Scale of the Nurse Work Index, consisting of five subscales: foundations of quality of care, nurse participation in hospital affairs, nurse manager, leadership and support of nurses, staffing and resource adequacy, and collegial relationships. The work environment was classified as positive when the mean score was 2.5 or above [19]. Missing supplies or broken equipment and insufficient staffing were measured using a 4‐point Likert scale (1 = frequently, 4 = never; responses 3‐4 were dichotomized as positive). Working time with COVID‐19 patients and incidence of death and dying was measured using a 4‐point Likert scale (1 = never, 4 = routinely; responses 1‐2 were dichotomized as positive). These two questions were specifically included because data were collected during the COVID‐19 pandemic, when healthcare workers were exposed to the virus and experienced an increased incidence of patient deaths.

### 2.6. Data Analysis

Data were analysed using SPSS Version 30 (IBM Corporation, 2024). Surveys with missing sections of data were not included; however, surveys with a few questions missing from a section were included. Missing data were left blank for items, but mean imputation was used for scales. Descriptive statistics (frequencies, percentages, means and standard deviations) were computed to compare nurse characteristics, nurse and patient outcomes, and organisational factors in national, private and public hospitals. Primary units of measurement were nurses (registered nurses, enrolled nurses and auxiliary nurses) (see Tables 1 and 2).

**TABLE 1:** Presents characteristics of respondents.

**Demographic variable**	**Grouping**	**National**		**Public**		**Private**	
		***n***	**%**	***n***	**%**	***n***	**%**

Province	Eastern Cape	351	8.2	310	14.0	41	2.0
	Free State	270	6.3	203	9.2	67	3.2
	Gauteng	1252	29.3	318	14.4	934	45.2
	Kwa‐Zulu Natal	679	15.9	265	12.0	414	20.0
	Limpopo	304	7.1	224	10.1	80	3.9
	Mpumulanga	246	5.7	208	9.4	38	1.8
	North West	243	5.7	235	10.6	8	0.4
	Northern Cape	237	5.5	202	9.1	35	1.7
	Western Cape	698	16.3	248	11.2	450	21.8

Specialty area of unit	Medical	860	20.0	467	21.1	393	18.8
	Surgical	939	21.8	483	21.8	456	21.9
	Trauma	332	7.7	176	8.0	156	7.5
	Maternity	449	10.4	289	13.1	160	7.7
	Critical Care	716	16.7	235	10.6	481	23.1
	Theatre	332	7.7	135	6.1	197	9.4
	Paediatrics	399	9.3	270	12.2	129	6.2
	Neonatal Intensive Care Unit	123	2.9	54	2.4	69	3.3
	Oncology	30	0.7	11	0.5	19	0.9
	Burns	9	0.2	9	0.4	0	0
	COVID‐19	70	1.6	67	3.0	3	0.1
	Other	39	0.9	17	0.8	22	1.1

Employment status	Full‐time	3906	91.3	1965	89.4	1941	93.3
	Part‐time	87	2.0	61	2.8	26	1.2
	Temporary/Agency	286	6.6	172	7.8	114	5.4

Nurse category	Registered nurse/midwife	2494	58.3	1165	52.9	1329	64.0
	Community service nurse	77	1.8	69	3.1	8	0.4
	Enrolled nurse	752	17.6	423	19.2	329	15.8
	Auxiliary nurse	955	22.3	544	24.7	411	19.8

Bachelor’s degree	Yes	780	32.5	389	35.8	391	29.8
	No	1620	67.5	699	64.2	921	70.2

**Demographic variable**	**Range**	**M**	**SD**	**M**	**SD**	**M**	**SD**

Age		42.5	10.2	41.8	10.2	43.2	10.1
Work experience		15.1	10.8	15.8	11.4	14.4	10.2

**TABLE 2:** Presents descriptive statistics for nurse‐reported outcomes.

Outcomes	National	Public	Private
	%	%	%
*Nurse outcomes*			
Job dissatisfaction	32.1	42.7	20.6
Intention to leave job	25.6	27.2	23.9
High burnout	43.9	49.0	34.7
Poor mental health	75.8	82.6	66.8
Poor physical health	88.1	93.3	81.2

*Patient Outcomes*			
Not recommend hospital to family and friends	24.9	32.2	14.9
Not confident that patients and their caregivers can manage their care after discharge	42.1	48.9	29.7
Not confident that managers will act to resolve problems in patient care	51.5	39.5	39.1
Poor/fair quality of care	30.1	37.4	20.0
Poor/failing patient safety grade	38.6	48.0	25.7

*Modifiable Organisational Factors*			
Unfavourable Practice Environment: overall	46.8	55.6	36.1
Unfavourable: Foundations of quality care	27.5	34.0	19.5
Unfavourable: Nurse participation in hospital affairs	56.4	68.8	41.2
Unfavourable: Nurse manager, leadership and support of nurses	48.2	58.7	35.4
Unfavourable: Staffing and resource adequacy	66.9	73.4	58.9
Unfavourable: Collegial relationships	39.8	41.1	38.2
Routine care for COVID patients	45.3	46.5	43.9
Routine care for patients experiencing death and dying	28.7	35.6	21.1
Missing supplies or broken equipment	67.3	23.0	54.4
Insufficient staffing	82.6	86.5	77.4

To estimate the association of organisational factors (working time with COVID‐19 patients, incidence of death and dying, resources and staffing and the practice environment) with negatively scaled nurse outcomes (job dissatisfaction, intention to leave, burnout, poor mental health and poor physical health) and patient outcomes (not recommending the hospital to family and friends, not being confident patients can manage care, not being confident management can resolve patient problems, poor/fair ward quality and poor/failing safety grade), nurse responses were aggregated to the hospital level, and the hospital was used as the primary unit of measurement using robust logistic regression models. Primary units of measurement were hospitals (see Tables 3 and 4).

**TABLE 3:** Odds ratios between nurse and patient outcomes, and organisational factors by public and private hospital and overall.

Outcomes	Effect	National		Public		Private	
		OR	[95% CI]	OR	[95% CI]	OR	[95% CI]
Job dissatisfaction	Practice Environment	4.53^∗∗^	(3.75 to 5.47)	3.47^∗∗^	(2.69 to 4.47)	5.29^∗∗^	(4.15 to 6.74)
	COVID‐19	1.19^∗^	(1.00 to 1.42)	1.09	(0.88 to 1.35)	1.35^∗^	(1.02 to 1.78)
	Death and Dying	1.34^∗∗^	(1.16 to 1.56)	1.10	(0.92 to 1.32)	1.21	(0.93 to 1.57)
	Resources	2.32^∗∗^	(1.94 to 2.76)	1.88^∗∗^	(1.44 to 2.44)	1.95^∗∗^	(1.53 to 2.48)
	Staffing	2.69^∗∗^	(2.12 to 3.42)	2.14^∗∗^	(1.58 to 2.92)	2.94^∗∗^	(1.99 to 4.36)

Intention to leave	Practice Environment	2.62^∗∗^	(2.13 to 3.23)	2.02^∗∗^	(1.50 to 2.73)	3.62^∗∗^	(2.79 to 4.70)
	COVID‐19	1.15	(0.95 to 1.39)	1.00	(0.80 to 1.26)	1.34	(0.99 to 1.80)
	Death and Dying	1.23^∗^	(1.04 to 1.46)	1.33^∗^	(1.11 to 1.59)	1.02	(0.75 to 1.39)
	Resources	1.55^∗∗^	(1.31 to 1.84)	1.46^∗^	(1.13 to 1.88)	1.58^∗∗^	(1.21 to 2.07)
	Staffing	2.20^∗∗^	(1.74 to 2.79)	1.62^∗∗^	(1.20 to 2.19)	2.97^∗∗^	(2.06 to 4.27)

Burnout	Practice Environment	4.75^∗∗^	(4.11 to 5.49)	3.96^∗∗^	(3.33 to 4.71)	5.17^∗∗^	(4.08 to 6.55)
	COVID‐19	1.32^∗^	(1.13 to 1.55)	1.31^∗^	(1.08 to 1.60)	1.32^∗^	(1.04 to 1.67)
	Death and Dying	1.68^∗∗^	(1.44 to 1.94)	1.62^∗∗^	(1.35 to 1.95)	1.38^∗^	(1.08 to 1.77)
	Resources	2.19^∗∗^	(1.83 to 2.63)	1.80^∗∗^	(1.33 to 2.43)	2.08^∗∗^	(1.72 to 2.52)
	Staffing	3.26^∗∗^	(2.52 to 4.22)	2.30^∗∗^	(1.60 to 3.32)	4.36^∗∗^	(3.11 to 6.12)

Poor mental health	Practice Environment	2.78^∗∗^	(2.38 to 3.23)	2.50^∗∗^	(2.01 to 3.10)	2.42^∗∗^	(1.95 to 3.00)
	COVID‐19	1.02	(0.87 to 1.20)	1.14	(0.89 to 1.44)	1.05	(0.84 to 1.30)
	Death and Dying	1.24^∗^	(1.03 to 1.48)	1.14	(0.87 to 1.49)	1.02	(0.81 to 1.29)
	Resources	2.24^∗∗^	(1.87 to 2.69)	1.60^∗∗^	(1.20 to 2.13)	2.16^∗∗^	(1.75 to 2.67)
	Staffing	2.36^∗∗^	(1.96 to 2.84)	1.42^∗^	(1.13 to 1.79)	2.79^∗∗^	(2.21 to 3.50)

Poor physical health	Practice Environment	3.44^∗∗^	(2.63 to 4.50)	3.70^∗∗^	(2.15 to 6.38)	2.45^∗∗^	(1.85 to 3.26)
	COVID‐19	1.01	(0.80 to 1.26)	1.19	(0.79 to 1.80)	1.06	(0.83 to 1.34)
	Death and Dying	1.42^∗^	(1.10 to 1.85)	1.2	(0.83 to 1.72)	1.17	(0.82 to 1.67)
	Resources	2.47^∗∗^	(1.97 to 3.11)	1.56	(0.97 to 2.50)	2.17^∗∗^	(1.64 to 2.87)
	Staffing	3.37^∗∗^	(2.68 to 4.24)	2.15^∗^	(1.14 to 4.06)	3.43^∗∗^	(2.69 to 4.39)

Not recommending hospital to family and friends	Practice Environment	5.11^∗∗^	(4.26 to 6.12)	3.69^∗∗^	(2.97 to 4.59)	7.27^∗∗^	(5.47 to 9.68)
	COVID‐19	1.13	(0.95 to 1.34)	1.12	(0.89 to 1.40)	1.10	(0.86 to 1.40)
	Death and Dying	1.43^∗∗^	(1.21 to 1.70)	1.34^∗^	(1.10 to 1.64)	1.03	(0.76 to 1.39)
	Resources	2.49^∗∗^	(2.02 to 3.06)	1.73^∗∗^	(1.30 to 2.30)	2.73^∗∗^	(2.05 to 3.36)
	Staffing	2.40^∗∗^	(1.84 to 3.13)	1.80^∗∗^	(1.27 to 2.55)	2.95^∗∗^	(1.92 to 4.54)

Not confident patients can manage care	Practice Environment	6.43^∗∗^	(5.53 to 7.47)	2.91^∗∗^	(2.38 to 3.55)	4.85^∗∗^	(3.87 to 6.08)
	COVID‐19	1.05	(1.24 to 0.89)	1.05	(0.89 to 1.24)	1.07	(0.85 to 1.34)
	Death and Dying	1.52^∗∗^	(1.29 to 1.78)	1.36^∗∗^	(1.15 to 1.60)	1.35^∗^	(1.03 to 1.78)
	Resources	3.02^∗∗^	(2.59 to 3.53)	1.90^∗∗^	(1.48 to 2.44)	2.12^∗∗^	(1.72 to 2.60)
	Staffing	3.68^∗∗^	(2.96 to 4.58)	1.72^∗∗^	(1.35 to 2.20)	2.19^∗∗^	(1.74 to 2.75)

Not confident management can resolve patient problems	Practice Environment	5.60^∗∗^	(4.64 to 6.77)	5.76^∗∗^	(4.54 to 7.31)	6.14^∗∗^	(5.00 to 7.53)
	COVID‐19	1.20	(1.02 to 1.41)	1.15	(0.93 to 1.41)	1.30^∗^	(1.01 to 1.69)
	Death and Dying	1.58^∗∗^	(1.32 to 1.88)	1.25^∗^	(1.02 to 1.54)	1.51^∗^	(1.14 to 1.99)
	Resources	3.11^∗∗^	(2.58 to 3.75)	2.58^∗∗^	(2.10 to 3.18)	2.63^∗^	(2.09 to 3.31)
	Staffing	3.33^∗∗^	(2.46 to 4.52)	2.52^∗∗^	(1.85 to 3.42)	5.08^∗∗^	(3.72 to 6.92)

Poor/fair ward quality	Practice Environment	3.92^∗∗^	(3.33 to 4.60)	4.16^∗∗^	(3.34 to 5.19)	7.47^∗∗^	(5.42 to 10.31)
	COVID‐19	1.01	(0.88 to 1.16)	1.1	(0.92 to 1.31)	1.39^∗^	(1.04 to 1.87)
	Death and Dying	1.53^∗∗^	(1.31 to 1.78)	1.62^∗^	(1.10 to 1.34)	1.57^∗^	(1.16 to 2.12)
	Resources	2.37^∗∗^	(2.00 to 2.81)	2.28^∗∗^	(1.80 to 2.88)	3.36^∗∗^	(2.56 to 4.42)
	Staffing	2.13^∗∗^	(1.80 to 2.51)	2.22^∗∗^	(1.59 to 3.09)	5.58^∗∗^	(3.55 to 8.77)

Poor/failing safety grade	Practice Environment	5.07^∗∗^	(4.42 to 5.82)	4.16^∗∗^	(3.55 to 4.87)	5.43^∗∗^	(4.36 to 6.76)
	COVID‐19	1.17	(1.00 to 1.37)	1.09	(0.91 to 1.31)	1.28	(0.96 to 1.71)
	Death and Dying	1.54^∗∗^	(1.30 to 1.83)	1.35^∗∗^	(1.11 to 1.64)	1.37^∗^	(1.04 to 1.79)
	Resources	2.67^∗∗^	(2.20 to 3.23)	2.37^∗∗^	(1.83 to 3.08)	2.08^∗∗^	(1.61 to 2.68)
	Staffing	3.23^∗∗^	(2.55 to 4.10)	2.63^∗∗^	(1.92 to 3.61)	3.48^∗∗^	(2.52 to 4.81)

^∗^Statistically significant at the 0.05 level (2‐tailed).

^∗∗^Statistically significant at the 0.01 level (2‐tailed).

**TABLE 4:** Odds ratios between the subscales of the practice environment and nurse and patient outcomes by public and private hospital and overall.

Outcomes	Effect	National		Public		Private	
		OR	[95% CI]	OR	[95% CI]	OR	[95% CI]
Job dissatisfaction	PES‐NWI:Foundations of Quality of Care	3.48^∗∗^	(2.96 to 4.10)	2.56^∗∗^	(2.14 to 3.05)	4.59^∗∗^	(3.47 to 6.07)
	PES‐NWI:Nurse participation in hospital affairs	4.67^∗∗^	(4.08 to 5.36)	3.47^∗∗^	(2.91 to 4.14)	4.71^∗∗^	(3.83 to 5.79)
	PES‐NWI: Nurse manager, leadership and support of nurses	4.71^∗∗^	(3.97 to 5.58)	3.15^∗∗^	(2.57 to 3.86)	6.46^∗∗^	(5.12 to 8.14)
	PES‐NWI: Staffing and resource adequacy	3.46^∗∗^	(2.84 to 4.20)	2.81^∗∗^	(2.18 to 3.61)	3.74^∗∗^	(2.82 to 4.95)
	PES‐NWI: Collegial relationships	2.68^∗∗^	(2.33 to 3.08)	2.41^∗∗^	(2.04 to 2.83)	3.50^∗∗^	(2.76 to 4.43)

Intention to leave	PES‐NWI: Foundations of Quality of Care	2.17^∗∗^	(1.77 to 2.66)	1.73^∗∗^	(1.36 to 2.21)	3.06^∗∗^	(2.34 to 4.01)
	PES‐NWI: Nurse participation in hospital affairs	2.48^∗∗^	(2.04 to 3.02)	1.73^∗∗^	(1.32 to 2.28)	3.69^∗∗^	(2.85 to 4.79)
	PES‐NWI: Nurse manager, leadership and support of nurses	2.81^∗∗^	(2.33 to 3.38)	2.12^∗∗^	(1.67 to 2.70)	4.01^∗∗^	(3.09 to 5.22)
	PES‐NWI: Staffing and resource adequacy	2.04^∗∗^	(1.64 to 2.54)	1.61^∗^	(1.18 to 2.19)	2.54^∗∗^	(1.88 to 3.45)
	PES‐NWI: Collegial relationships	2.01^∗∗^	(1.69 to 2.41)	1.54^∗∗^	(1.22 to 1.94)	2.86^∗∗^	(2.27 to 3.61)

Burnout	PES‐NWI: Foundations of Quality of Care	3.53^∗∗^	(2.99 to 4.16)	2.87^∗∗^	(2.37 to 3.47)	4.08^∗∗^	(3.14 to 5.30)
	PES‐NWI: Nurse participation in hospital affairs	4.05^∗∗^	(3.49 to 4.69)	3.30^∗∗^	(2.62 to 4.16)	4.20^∗∗^	(3.50 to 5.04)
	PES‐NWI: Nurse manager, leadership and support of nurses	3.88^∗∗^	(3.34 to 4.52)	3.37^∗∗^	(2.66 to 4.27)	3.84^∗∗^	(3.14 to 4.69)
	PES‐NWI: Staffing and resource adequacy	3.94^∗∗^	(3.30 to 4.71)	3.00^∗∗^	(2.38 to 3.77)	4.79^∗∗^	(3.89 to 5.89)
	PES‐NWI: Collegial relationships	3.16^∗∗^	(2.77 to 3.61)	3.10^∗∗^	(2.58 to 3.73)	3.35^∗∗^	(2.72 to 4.13)

Poor mental health	PES‐NWI: Foundations of Quality of Care	2.37^∗∗^	(1.98 to 2.84)	2.06^∗∗^	(1.64 to 2.60)	2.13^∗∗^	(1.58 to 2.86)
	PES‐NWI: Nurse participation in hospital affairs	2.52^∗∗^	(2.19 to 2.90)	1.96^∗∗^	(1.55 to 2.47)	2.28^∗∗^	(1.87 to 2.79)
	PES‐NWI: Nurse manager, leadership and support of nurses	2.58^∗∗^	(2.19 to 3.04)	2.30^∗∗^	(1.83 to 2.90)	2.15^∗∗^	(1.72 to 2.68)
	PES‐NWI: Staffing and resource adequacy	2.56^∗∗^	(2.17 to 3.03)	1.83^∗∗^	(1.47 to 2.29)	2.77^∗∗^	(2.26 to 3.40)
	PES‐NWI: Collegial relationships	2.23^∗∗^	(1.91 to 2.61)	2.44^∗∗^	(1.86 to 3.21)	2.07^∗∗^	(1.74 to 2.46)

Poor physical health	PES‐NWI: Foundations of Quality of Care	2.40^∗∗^	(1.76 to 3.28)	1.91^∗^	(1.18 to 3.09)	2.05^∗∗^	(1.35 to 3.11)
	PES‐NWI: Nurse participation in hospital affairs	**3**.**31** ^∗∗^	(2.61 to 4.19)	2.91^∗∗^	(1.79 to 4.73)	2.39^∗∗^	(1.84 to 3.11)
	PES‐NWI: Nurse manager, leadership and support of nurses	2.86^∗∗^	(2.17 to 3.75)	2.90^∗∗^	(1.67 to 5.03)	1.97^∗∗^	(1.49 to 2.60)
	PES‐NWI: Staffing and resource adequacy	3.23^∗∗^	(2.65 to 3.92)	2.28^∗∗^	(1.60 to 3.23)	3.11^∗∗^	(2.46 to 3.92)
	PES‐NWI: Collegial relationships	2.46^∗∗^	(1.90 to 3.20)	3.41^∗∗^	(2.14 to 5.45)	2.09^∗∗^	(1.56 to 2.79)

Not recommend hospital to family and friends	PES‐NWI: Foundations of Quality of Care	4.43^∗∗^	(3.66 to 5.36)	3.15^∗∗^	(2.64 to 3.77)	7.05^∗∗^	(4.99 to 9.96)
	PES‐NWI: Nurse participation in hospital affairs	4.95^∗∗^	(4.08 to 6.01)	3.29^∗∗^	(2.82 to 3.84)	6.47^∗∗^	(4.58 to 9.12)
	PES‐NWI: Nurse manager, leadership and support of nurses	5.13^∗∗^	(4.29 to 6.13)	3.75^∗∗^	(3.19 to 4.42)	6.42^∗∗^	(4.51 to 9.13)
	PES‐NWI: Staffing and resource adequacy	3.40^∗∗^	(2.78 to 4.15)	2.56^∗∗^	(2.07 to 3.15)	4.40^∗∗^	(3.01 to 6.41)
	PES‐NWI: Collegial relationships	2.95^∗∗^	(2.45 to 3.56)	2.55^∗∗^	(1.95 to 3.32)	4.45^∗∗^	(3.20 to 6.19)

Not confident patients can manage care	PES‐NWI: Foundations of Quality of Care	4.26^∗∗^	(3.57 to 5.08)	2.66^∗∗^	(2.18 to 3.26)	5.05^∗∗^	(3.88 to 6.59)
	PES‐NWI: Nurse participation in hospital affairs	6.51^∗∗^	(5.79 to 7.32)	3.01^∗∗^	(2.45 to 3.69)	3.52^∗∗^	(2.85 to 4.35)
	PES‐NWI: Nurse manager, leadership and support of nurses	5.23^∗∗^	(4.44 to 6.15)	2.57^∗∗^	(2.14 to 3.09)	3.42^∗∗^	(2.73 to 4.29)
	PES‐NWI: Staffing and resource adequacy	5.09^∗∗^	(4.36 to 5.94)	2.25^∗∗^	(1.81 to 2.79)	4.63^∗∗^	(3.54 to 6.05)
	PES‐NWI: Collegial relationships	2.85^∗∗^	(2.46 to 3.30)	2.16^∗∗^	(1.82 to 2.57)	3.17^∗∗^	2.58 to 3.88)

Not confident management can resolve patient problems	PES‐NWI: Foundations of Quality of Care	4.88^∗∗^	(4.02 to 5.93)	3.60^∗∗^	(2.91 to 4.45)	4.36^∗∗^	(3.16 to 6.03)
	PES‐NWI: Nurse participation in hospital affairs	4.54^∗∗^	(3.75 to 5.50)	5.76^∗∗^	(4.70 to 7.06)	5.92^∗∗^	(4.97 to 7.04)
	PES‐NWI: Nurse manager, leadership and support of nurses	4.25^∗∗^	(3.52 to 5.14)	4.50^∗∗^	(3.48 to 5.81)	5.01^∗∗^	(4.07 to 6.15)
	PES‐NWI: Staffing and resource adequacy	4.67^∗∗^	(3.78 to 5.76)	4.30^∗∗^	(3.56 to 5.20)	5.40^∗∗^	(4.05 to 7.20)
	PES‐NWI: Collegial relationships	3.14^∗∗^	(2.62 to 3.76)	3.01^∗∗^	(2.42 to 3.74)	2.81^∗∗^	(2.29 to 3.45)

Poor/fair ward quality	PES‐NWI: Foundations of Quality of Care	3.67^∗∗^	(3.09 to 4.36)	3.90^∗∗^	(3.05 to 4.98)	5.94^∗∗^	(4.42 to 7.98)
	PES‐NWI: Nurse participation in hospital affairs	3.74^∗∗^	(3.22 to 4.33)	3.58^∗∗^	(2.83 to 4.52)	4.60^∗∗^	(3.54 to 5.96)
	PES‐NWI: Nurse manager, leadership and support of nurses	3.28^∗∗^	(2.83 to 3.79)	3.20^∗∗^	(2.58 to 3.96)	5.07^∗∗^	(3.85 to 6.66)
	PES‐NWI: Staffing and resource adequacy	3.27^∗∗^	(2.72 to 3.93)	3.27^∗∗^	(2.61 to 4.09)	7.24^∗∗^	(4.79 to 10.95)
	PES‐NWI: Collegial relationships	2.47^∗∗^	(2.17 to 2.81)	2.95^∗∗^	(2.28 to 3.81)	3.73^∗∗^	(2.81 to 4.95)

Poor/failing safety grade	PES‐NWI: Foundations of Quality of Care	4.21^∗∗^	(3.51 to 5.06)	3.63^∗∗^	(2.94 to 4.49)	4.16^∗∗^	(3.17 to 5.47)
	PES‐NWI: Nurse participation in hospital affairs	4.35^∗∗^	(3.81 to 4.95)	3.54^∗∗^	(3.04 to 4.12)	3.98^∗∗^	(3.23 to 4.91)
	PES‐NWI: Nurse manager, leadership and support of nurses	3.48^∗∗^	(2.99 to 4.04)	2.86^∗∗^	(2.40 to 3.41)	3.31^∗∗^	(2.73 to 4.02)
	PES‐NWI: Staffing and resource adequacy	4.28^∗∗^	(3.62 to 5.05)	3.27^∗∗^	(2.71 to 3.94)	5.37^∗∗^	(3.87 to 7.36)
	PES‐NWI: Collegial relationships	2.62^∗∗^	(2.27 to 3.02)	2.46^∗∗^	(2.05 to 2.95)	3.10^∗∗^	(2.43 to 3.96)

^∗^Statistically significant at the 0.05 level (2‐tailed).

^∗∗^Statistically significant at the 0.01 level (2‐tailed).

In analysing these outcomes and organisational factors, hospital type (private/public) was significant in the odds ratios (*p* ≤ 0.001), and therefore findings are reported separately as national, public and private. Hospital levels (central/tertiary, provincial and district [based on hospital size and services offered]), private hospital groups, unit types (specialty areas), and nurse characteristics (including employment status, nurse category, education, age, and work experience) were statistically significant; however, the effect sizes were small and not considered meaningful. Therefore, only unadjusted odds ratios are reported, while adjusted odds ratios are provided as supporting information (see Supporting File 1). Reporting within this study is done in accordance with the STROBE statement.

### 2.7. Role of the Funding Source

The funder was not involved in the study design, in the collection, analysis, and interpretation of data, in the writing of the report, or in the decision to submit the paper for publication.

### 2.8. Ethical Approval

Ethics approval was granted by the North‐West University Health Research Ethics Committee (NWU‐00033‐19‐A1). In the private sector, approval was granted by the ethics committees of the private hospital groups. In the public sector, approval was granted at national, provincial and district levels for each individual hospital.

## 3. Results

Most respondents were from surgical (*n* = 939; 21.8%), medical (*n* = 860; 20%), and critical care units (*n* = 716; 16.7%) and employed full‐time (*n* = 3906; 91.3%). Most were registered nurses (*n* = 2494; 58.3%), with 780 (32.5%) of registered nurses having a bachelor’s degree. They were 42.5 years of age (SD = 10.5) on average and had 15.1 years (SD = 10.8) of experience.

As shown in Table 2, one‐third of nurses were dissatisfied with their jobs (*n* = 1325, 32.1%), with one‐quarter intending to leave their jobs (*n* = 1078, 25.6%). Of nurses, 43.9% (*n* = 1673) experienced high levels of burnout, with 75.8% (*n* = 2703) and 88.1% (*n* = 3074) exhibiting mental and physical health symptoms, respectively. Nurse outcomes were consistently reported to be worse in the public sector than in the private sector (*p* ≤ 0.001).

Regarding patient outcomes, half of the nurses (*n* = 1904, 51.5%) were only somewhat confident or not confident at all that management will act to resolve problems in patient care. One‐third of nurses (*n* = 1113, 30.1%) reported the quality of nursing care in their units as fair/poor, and more nurses (*n* = 1429, 38.6%) rated overall patient safety as poor/failing. Again, public sector nurses rated patient outcomes as poorer than those from the private sector (*p* ≤ 0.001).

Almost half (*n* = 1810, 46.8%) of the nurses rated their overall practice environment as unfavourable, with nurses (*n* = 2582, 66.9%) agreeing on staffing and resource inadequacy, making this the most unfavourable aspect of the practice environment in both sectors. Congruent with previous findings, the public sector practice environment was perceived by more as unfavourable than in the private sector (*p* ≤ 0.001).

Almost half (*n* = 1790, 45.3%) of the nurses had to occasionally or routinely care for COVID‐19 patients, with 28.7% (*n* = 1107) facing death and dying occasionally or routinely. Most nurses (*n* = 2424, 67.3% and *n* = 2962, 82.6%) were frequently or occasionally faced with missing supplies or insufficient staffing, respectively.

As shown in Table 3, the organisational factors associated with nurse and patient outcomes were the practice environment, staffing, and resources. Routine care for patients experiencing death and dying and working time with COVID‐19 patients, for the most part, was not significant. Regarding nurse outcomes, job dissatisfaction (OR = 4.53^∗∗^; CI 3.75–5.47) and burnout (OR = 4.75^∗∗^; CI 4.11 –5.49) are five times less likely, and intention to leave (OR = 2.62^∗∗^; CI 2.13–3.23) poor mental health (OR = 4.53; CI 3.75–5.47) and poor physical health (OR = 2.78^∗∗^; CI 2.38–3.23) are three times less likely in a favourable practice environment. Regarding patient outcomes, in a favourable practice environment, not being confident that patients can manage care (OR = 6.43^∗∗^; CI 5.53–7.47) is seven times less likely; not being confident that management can resolve patient problems (OR = 5.60^∗∗^; CI 4.64–6.77) is six times less likely; not recommending the hospital to family and friends (OR = 5.11; CI 4.26–6.12) and a poor/failing safety grade (OR = 5.07^∗∗^; CI 4.42–5.82) are five times less likely, while poor/fair ward quality (OR = 3.92^∗∗^; CI 3.33–4.60) is four times less likely.

Given that the practice environment demonstrated the strongest overall association with nurse and patient outcomes, the authors conducted a detailed analysis to identify which specific subscales within the practice environment were most strongly associated with these outcomes. Regarding nurse outcomes, job dissatisfaction (OR = 4.571^∗∗^; CI 3.97–5.58) was five times less likely, and intention to leave (OR = 2.81^∗∗^; CI 2.33–3.38) and poor mental health (OR = 2.58^∗∗^; CI 2.19–3.04) were three times less likely when the subscale of nurse manager, leadership and support of nurses was positive. When the subscale of nurse participation in hospital affairs was positive, burnout (OR = 4.05^∗∗^; CI 3.49–4.69) was four times less likely and poor physical health (OR = 3.31^∗∗^; CI 2.61–4.19) was three times less likely.

Regarding patient outcomes, not recommending the hospital to family and friends (OR = 5.13^∗∗^; CI 4.29–6.13) was five times less likely when the subscale of nurse manager, leadership and support of nurses was positive. When the subscale of nurse participation in hospital affairs was positive, not being confident that patients can manage care (OR = 6.51^∗∗^; CI 5.79–7.32) was seven times less likely, while poor/fair ward quality (OR = 3.74^∗∗^; CI 3.22–4.33) and poor/failing safety grade (OR = 4.35^∗∗^; CI 3.81–4.95) were four times less likely. Finally, not being confident that management can resolve patient problems (OR = 4.88^∗∗^; CI 4.02–5.93) is five times less likely when the subscale of foundations of quality of care is positive.

## 4. Discussion

Our results in SA re‐enforce the WHO‐Europe report *Time to Act* that emphasises the need for all countries to prioritise in their workforce policies interventions targeted at improving clinician retention. *Time to Act* concludes that no country can solve its shortages of health professionals by training more as long as retention is poor. Our study of South African hospitals finds that approximately one‐third of nurses were dissatisfied with their jobs, and about one‐quarter indicated an intention to leave their current positions. Nearly half of hospital nurses experienced high levels of burnout, while over three‐quarters reported negative symptoms affecting both their mental and physical health. Although this study was conducted during the COVID‐19 pandemic, the findings remain consistent with those of a national study conducted nearly a decade earlier [20]. This suggests that the pandemic, which has since passed, is unlikely to be the primary explanation for nurse retention challenges; instead, persistent deficiencies in hospital work environments appear to be the key contributing factor. The good news is that work environments are feasible to improve.

Patient outcomes appear to have deteriorated further since the earlier study [20], which may reflect the timing of data collection during the COVID‐19 pandemic. Fewer than half of the nurses expressed confidence in patients’ and caregivers’ ability to manage care following discharge, and more than half lacked confidence that hospital management would effectively address issues in patient care. Additionally, 25% of nurses reported that they would not recommend their hospital to family or friends. Approximately one‐third rated the quality of ward‐based care as poor or fair and assigned a poor or failing grade to overall patient safety. Notably, both nurse and patient outcomes were significantly poorer in the public sector compared to the private sector.

When determining the main organisational factors that impacted on nurse and patient outcomes during the COVID‐19 pandemic, our results provide empirical evidence that favourable practice environments and adequate staffing and resources are associated with better nurse and patient outcomes. The practice environment was experienced as unfavourable by more than half of the nurses, with the subscale of staffing and resource adequacy experienced most negatively. It is well established through a recent meta‐analysis that favourable practice environments are associated with lower odds of negative nurse outcomes (job dissatisfaction burnout and intention to leave) and patient outcomes (confidence that their patients could manage care after discharge, fair/poor ward quality and poor/failing safety grade) [19]. So also, systematic reviews on nurse staffing show strong associations between nurse [21] and patient outcomes [22]. Other recent studies conducted postpandemic in the United States of America and Europe had comparable results [23, 24].

Organisational factors of working time with COVID‐19 patients and routine care for patients experiencing death and dying had a minimal effect on nurse and patient outcomes, a similar finding to Aiken et al. [25], who found that nurses who worked in favourable practice environments prior to the pandemic reported significantly better outcomes during the pandemic, with only a 3% increase in the prevalence of burnout [25].

A focused analysis of the practice environment revealed that two subscales—nurse manager leadership and support of nurses, and nurse participation in hospital affairs—had the strongest associations with both nurse and patient outcomes. These findings align with those of a recent integrative review, which identified leadership as the most critical factor enabling nurses to practise effectively in hospital settings, followed by autonomy in decision‐making, teamwork, adequate staffing and resources, and organisational commitment to supporting nurses [26].

Repeated major systematic reviews have consistently demonstrated that relationship‐oriented leadership is strongly associated with improved nurse outcomes [27, 28], enhanced patient outcomes [29], and a more favourable practice environment [27].

Strengthening senior nurse leadership has considerable potential to improve healthcare governance and management frameworks by enabling more active and informed participation of nurses in institutional decision‐making, influencing health policy development, and contributing to strategic workforce planning. The importance of leadership development has also been emphasised in key global and national policy documents. The World Health Organization’s State of the World’s Nursing report [3], along with national directives [1, 2], has specifically highlighted the need to strengthen leadership competencies among nurses as a critical strategy to improve health system performance and workforce sustainability.

In many South African hospitals, however, governance structures remain characterised by hierarchical and exclusionary leadership models that limit nurses’ involvement in operational and policy decisions. This lack of inclusion has contributed to a decision‐making gap with adverse implications for both nurse and patient outcomes [30]. Addressing this gap requires investment in leadership development across the nursing workforce, including early‐career nurses. Leadership development programmes have demonstrated benefits for both nurses and health systems, and when targeted at early‐career nurses, they also support workforce retention and sustained professional development [27]. Strengthening leadership capacity across the profession may also enable nurses to advocate for more inclusive organisational practices, such as shared governance structures, improved communication between hospital management and nurses, and greater involvement in quality improvement and policy development at both organisational and national levels.

The limitations of the study include its reliance on cross‐sectional data, which limits the ability to assert causality between the modifiable organisational factors of hospitals and nurse and patient outcomes. However, its strength lies in the fact that it is the first national study of nurses across SA since 2013 and that data were collected during and after the COVID‐19 pandemic.

## 5. Conclusion

This study provides compelling evidence that the practice environment is deficient in many South African hospitals and that improved retention of nurses and improved patient safety are contingent on improving organisational work environments in hospitals. The systemic challenges brought to the forefront by the COVID‐19 pandemic underscore the urgent need for structural reform within hospitals. The findings offer a focused, evidence‐informed foundation for targeted interventions. Prioritising leadership development, strengthening governance structures, enhancing management responsiveness to act on nurses’ recommendations for how to improve care, fostering greater engagement of nurses in decision‐making and policy formulation, and ensuring adequate staffing and resource availability are critical strategies. Collectively, these measures can enable South African healthcare institutions to formulate contextually relevant recruitment and retention strategies that promote workforce sustainability and improve the quality and safety of care delivered.

## Funding

This work was based on research supported in part by the National Research Foundation of South Africa (Grant Number 123541). Opinions expressed and conclusions arrived at, are those of the author and are not necessarily to be attributed to the NRF.

## Conflicts of Interest

The authors declare no conflicts of interest.

## Supporting Information

File 1: Odds ratios between nurse and patient outcomes, and organisational factors by public and private hospital and overall (Unadjusted and Adjusted Odds Ratios).

File 2: Odds ratios between the subscales of the practice environment and nurse and patient outcomes by public and private hospital and overall (Unadjusted and Adjusted Odds Ratios).

## Supporting information


**Supporting Information** Additional supporting information can be found online in the Supporting Information section.

## Data Availability

The data are not publicly available due to ethical approval restrictions.
