# Supplementary material for: Nurse and Patient Outcomes in Private and Public Hospitals in South Africa During the COVID‐19 Pandemic: A Cross‐Sectional Study
Source: J Nurs Manag. 2026 May 3;2026:1853384. doi: 10.1155/jonm/1853384 (PMC13136065; doi:10.1155/jonm/1853384)
Supplement: Supplementary file 1 — Supporting Information Additional supporting information can be found online in the Supporting Information section. [file JONM-2026-1853384-s001.docx]

Supplementary File

File 1: Odds ratios between nurse and patient outcomes, and organisational factors by public and private hospital and overall (Unadjusted and Adjusted Odds Ratios)

| **Outcome** | **Effect** | **NATIONAL** | | | | **PUBLIC** | | | | **PRIVATE** | | | |
| --- | --- | --- | --- | --- | --- | --- | --- | --- | --- | --- | --- | --- | --- |
|  |  | ***UNADJUSTED*** | | ***ADJUSTED*** | | ***UNADJUSTED*** | | ***ADJUSTED*** | | ***UNADJUSTED*** | | ***ADJUSTED*** | |
|  |  | **OR** | **[95% CI]** | **OR** | **[95% CI]** | **OR** | **[95% CI]** | **OR** | **[95% CI]** | **OR** | **[95% CI]** | **OR** | **[95% CI]** |
|  | | | | | | | | | | | | | |
| **Job dissatisfaction** | **Practice Environment** | 4∙53** | (3∙75 to 5∙47) | 4∙47** | (3∙67-5∙43) | 3∙47** | (2∙69 to 4∙47) | 3∙27** | (2∙48-4∙31) | 5∙29** | (4∙15 to 6∙74) | 5∙36** | (4∙20-6∙83) |
|  | **COVID-19** | 1∙19* | (1∙00 to 1∙42) | 1,19 | (0∙99-1∙42) | 1∙09 | (0∙88 to 1∙35) | 1,1 | (0∙89-1∙37) | 1∙35* | (1∙02 to 1∙78) | 1∙33* | (1∙00-1∙77) |
|  | **Death and Dying** | 1∙34** | (1∙16 to 1∙56) | 1∙37** | (1∙18-1∙60) | 1∙10 | (0∙92 to 1∙32) | 1,14 | (0∙95-1∙38) | 1∙21 | (0∙93 to 1∙57) | 1,22 | (0∙93-1∙61) |
|  | **Resources** | 2∙32** | (1∙94 to 2∙76) | 2∙37** | (1∙99-2∙84) | 1∙88** | (1∙44 to 2∙44) | 1∙87** | (1∙44-2∙44) | 1∙95** | (1∙53 to 2∙48) | 2∙03** | (1∙60-2∙58) |
|  | **Staffing** | 2∙69** | (2∙12 to 3∙42) | 2∙84** | (2∙23-3∙61) | 2∙14** | (1∙58 to 2∙92) | 2∙26** | (1∙66-3∙10) | 2∙94** | (1∙99 to 4∙36) | 2∙97** | (2∙00-4∙42) |
|  | | | | | | | | | | | | | |
| **Intention to leave** | **Practice Environment** | 2∙62** | (2∙13 to 3∙23) | 2∙70** | (2∙19-3∙33) | 2∙02** | (1∙50 to 2∙73) | 2∙06** | (1∙51-2∙80) | 3∙62** | (2∙79 to 4∙70) | 3∙76** | (2∙86-4∙95) |
|  | **COVID-19** | 1∙15 | (0∙95 to 1∙39) | 1,14 | (0∙93-1∙39) | 1∙00 | (0∙80 to 1∙26) | 1,01 | (0∙78-1∙30) | 1∙34 | (0∙99 to 1∙80) | 1,32 | (0∙97-1∙79) |
|  | **Death and Dying** | 1∙23* | (1∙04 to 1∙46) | 1∙22* | (1∙02-1∙45) | 1∙33* | (1∙11 to 1∙59) | 1∙29* | (1∙07-1∙55) | 1∙02 | (0∙75 to 1∙39) | 1,03 | (0∙74-1∙42) |
|  | **Resources** | 1∙55** | (1∙31 to 1∙84) | 1∙58** | (1∙32-1∙88) | 1∙46* | (1∙13 to 1∙88) | 1∙42* | (1∙10-1∙83) | 1∙58** | (1∙21 to 2∙07) | 1∙66** | (1∙26-2∙20) |
|  | **Staffing** | 2∙20** | (1∙74 to 2∙79) | 2∙22** | (1∙75-2∙83) | 1∙62** | (1∙20 to 2∙19) | 1∙53* | (1∙15-2∙05) | 2∙97** | (2∙06 to 4∙27) | 3∙06** | (2∙07-4∙51) |
|  | | | | | | | | | | | | | |
| **Burnout** | **Practice Environment** | 4∙75** | (4∙11 to 5∙49) | 4∙87** | (4∙21-5∙62) | 3∙96** | (3∙33 to 4∙71) | 4∙07** | (3∙42-4∙86) | 5∙17** | (4∙08 to 6∙55) | 5∙24** | (4∙15-6∙62) |
|  | **COVID-19** | 1∙32* | (1∙13 to 1∙55) | 1∙28* | (1∙09-1∙50) | 1∙31* | (1∙08 to 1∙60) | 1∙27* | (1∙05-1∙55) | 1∙32* | (1∙04 to 1∙67) | 1∙29* | (1∙02-1∙64) |
|  | **Death and Dying** | 1∙68** | (1∙44 to 1∙94) | 1∙64** | (1∙41-1∙90) | 1∙62** | (1∙35 to 1∙95) | 1∙63** | (1∙37-1∙93) | 1∙38* | (1∙08 to 1∙77) | 1∙33* | (1∙05-1∙69) |
|  | **Resources** | 2∙19** | (1∙83 to 2∙63) | 2∙25** | (1∙88-2∙69) | 1∙80** | (1∙33 to 2∙43) | 1∙93** | (1∙41-2∙62) | 2∙08** | (1∙72 to 2∙52) | 2∙04** | (1∙68-2∙48) |
|  | **Staffing** | 3∙26** | (2∙52 to 4∙22) | 3∙56** | (2∙74-4∙64) | 2∙30** | (1∙60 to 3∙32) | 2∙59** | (1∙71-3∙91) | 4∙36** | (3∙11 to 6∙12) | 4∙32** | (3∙06-6∙08) |
|  | | | | | | | | | | | | | |
| **Poor mental health** | **Practice Environment** | 2∙78** | (2∙38 to 3∙23) | 2∙77** | (2∙37-3∙24) | 2∙50** | (2∙01 to 3∙10) | 2∙53** | (2∙03-3∙16) | 2∙42** | (1∙95 to 3∙00) | 2∙41** | (1∙92-3∙03) |
|  | **COVID-19** | 1∙02 | (0∙87 to 1∙20) | 1,04 | (0∙88-1∙22) | 1∙14 | (0∙89 to 1∙44) | 1∙14 | (0∙88-1∙48) | 1∙05 | (0∙84 to 1∙30) | 1∙02 | (0∙82-1∙28) |
|  | **Death and Dying** | 1∙24* | (1∙03 to 1∙48) | 1∙21* | (1∙00-1∙46) | 1∙14 | (0∙87 to 1∙49) | 1∙14 | (0∙87-1∙50) | 1∙02 | (0∙81 to 1∙29) | 1 | (0∙78-1∙28) |
|  | **Resources** | 2∙24** | (1∙87 to 2∙69) | 2∙20** | (1∙86-2∙59) | 1∙60** | (1∙20 to 2∙13) | 1∙55** | (1∙19-2∙00) | 2∙16** | (1∙75 to 2∙67) | 2∙17** | (1∙78-2∙65) |
|  | **Staffing** | 2∙36** | (1∙96 to 2∙84) | 2∙39** | (1∙98-2∙87) | 1∙42* | (1∙13 to 1∙79) | 1∙40* | (1∙12-1∙76) | 2∙79** | (2∙21 to 3∙50) | 2∙85** | (2∙30-3∙54) |
|  | | | | | | | | | | | | | |
| **Poor physical health** | **Practice Environment** | 3∙44** | (2∙63 to 4∙50) | 3∙79* | (1∙65-8∙70) | 3∙70** | (2∙15 to 6∙38) | 3∙1* | (1∙25-7∙70) | 2∙45** | (1∙85 to 3∙26) | 2∙3 | (0∙61-8∙61) |
|  | **COVID-19** | 1∙01 | (0∙80 to 1∙26) | 1,13 | (0∙62-2∙06) | 1∙19 | (0∙79 to 1∙80) | 1,11 | (0∙52-2∙33) | 1∙06 | (0∙83 to 1∙34) | 1∙12 | (0∙42-2∙94) |
|  | **Death and Dying** | 1∙42* | (1∙10 to 1∙85) | 2∙83* | (1∙12-7∙13) | 1∙2 | (0∙83 to 1∙72) | 3,34 | (0∙62-17∙97) | 1∙17 | (0∙82 to 1∙67) | 1∙33 | (0∙36-4∙90) |
|  | **Resources** | 2∙47** | (1∙97 to 3∙11) | 3∙49** | (1∙88-6∙50) | 1∙56 | (0∙97 to 2∙50) | 1,63 | (0∙51-5∙22) | 2∙17** | (1∙64 to 2∙87) | 2∙95* | (1∙32-6∙58) |
|  | **Staffing** | 3∙37** | (2∙68 to 4∙24) | 3∙51** | (1∙77-6∙95) | 2∙15* | (1∙14 to 4∙06) | 1,64 | (0∙48-5∙58) | 3∙43** | (2∙69 to 4∙39) | 3∙55* | (1∙56-8∙09) |
|  | | | | | | | | | | | | | |
|  | | | | | | | | | | | | | |
| **Not confident patients can manage care** | **Practice Environment** | 6∙43** | (5∙53 to 7∙47) | 3∙98** | (3∙38-4∙67) | 2∙91** | (2∙38 to 3∙55) | 2∙93** | (2∙39-3∙61) | 4∙85** | (3∙87 to 6∙08) | 4∙84** | (3∙85-6∙09) |
|  | **COVID-19** | 1∙05 | (1∙24 to 0∙89) | 1,02 | (0∙89-1∙19) | 1∙05 | (0∙89 to 1∙24) | 1 | (0∙85-1∙19) | 1∙07 | (0∙85 to 1∙34) | 1,04 | (0∙82-1∙32) |
|  | **Death and Dying** | 1∙52** | (1∙29 to 1∙78) | 1∙50** | (1∙28-1∙77) | 1∙36** | (1∙15 to 1∙60) | 1∙38** | (1∙15-1∙65) | 1∙35* | (1∙03 to 1∙78) | 1,26 | (0∙95-1∙68) |
|  | **Resources** | 3∙02** | (2∙59 to 3∙53) | 2∙36** | (1∙99-2∙80) | 1∙90** | (1∙48 to 2∙44) | 1∙88** | (1∙45-2∙44) | 2∙12** | (1∙72 to 2∙60) | 2∙04** | (1∙63-2∙54) |
|  | **Staffing** | 3∙68** | (2∙96 to 4∙58) | 2∙19** | (1∙85-2∙58) | 1∙72** | (1∙35 to 2∙20) | 1∙71** | (1∙34-2∙18) | 2∙19** | (1∙74 to 2∙75) | 2∙18** | (1∙70-2∙80) |
|  | | | | | | | | | | | | | |
| **Not confident management can resolve patient problems** | **Practice Environment** | 5∙60** | (4∙64 to 6∙77) | 6∙31** | (5∙43-7∙35) | 5∙76** | (4∙54 to 7∙31) | 5∙44** | (4∙26-6∙95) | 6∙14** | (5∙00 to 7∙53) | 6∙12** | (4∙98-7∙52) |
|  | **COVID-19** | 1∙20 | (1∙02 to 1∙41) | 1,05 | (0∙89-1∙24) | 1∙15 | (0∙93 to 1∙41) | 1,12 | (0∙91-1∙38) | 1∙30* | (1∙01 to 1∙69) | 1,28 | (0∙99-1∙67) |
|  | **Death and Dying** | 1∙58** | (1∙32 to 1∙88) | 1∙47** | (1∙25-1∙74) | 1∙25* | (1∙02 to 1∙54) | 1,23 | (1∙00-1∙53) | 1∙51* | (1∙14 to 1∙99) | 1∙45* | (1∙09-1∙92) |
|  | **Resources** | 3∙11** | (2∙58 to 3∙75) | 3∙02** | (2∙58-3∙54) | 2∙58** | (2∙10 to 3∙18) | 2∙46** | (1∙99-3∙05) | 2∙63* | (2∙09 to 3∙31) | 2∙61** | (2∙08-3∙28) |
|  | **Staffing** | 3∙33** | (2∙46 to 4∙52) | 3∙65** | (2∙93-4∙54) | 2∙52** | (1∙85 to 3∙42) | 2∙37** | (1∙74-3∙23) | 5∙08** | (3∙72 to 6∙92) | 4∙90** | (3∙57-6∙72) |
|  | | | | | | | | | | | | | |
| **Poor/Fair ward quality** | **Practice Environment** | 3∙92** | (3∙33 to 4∙60) | 5∙65** | (4∙66-6∙86) | 4∙16** | (3∙34 to 5∙19) | 4∙19** | (3∙32-5∙30) | 7∙47** | (5∙42 to 10∙31) | 7∙42** | (5∙39-10∙20) |
|  | **COVID-19** | 1∙01 | (0∙88 to 1∙16) | 1∙20* | (1∙02-1∙41) | 1∙1 | (0∙92 to 1∙31) | 1∙1 | (0∙93-1∙32) | 1∙39* | (1∙04 to 1∙87) | 1∙36* | (1∙02-1∙82) |
|  | **Death and Dying** | 1∙53** | (1∙31 to 1∙78) | 1∙57** | (1∙31-1∙88) | 1∙62* | (1∙10 to 1∙34) | 1∙33* | (1∙10-1∙62) | 1∙57* | (1∙16 to 2∙12) | 1∙55* | (1∙15-2∙09) |
|  | **Resources** | 2∙37** | (2∙00 to 2∙81) | 3∙24** | (2∙70-3∙89) | 2∙28** | (1∙80 to 2∙88) | 2∙39** | (1∙92-2∙97) | 3∙36** | (2∙56 to 4∙42) | 3∙38** | (2∙57-4∙45) |
|  | **Staffing** | 2∙13** | (1∙80 to 2∙51) | 3∙46** | (2∙54-4∙71) | 2∙22** | (1∙59 to 3∙09) | 2∙30** | (1∙64-3∙22) | 5∙58** | (3∙55 to 8∙77) | 5∙44** | (3∙43-8∙61) |
|  | | | | | | | | | | | | | |
| **Poor/Failing safety  grade** | **Practice Environment** | 5∙07** | (4∙42 to 5∙82) | 5∙15** | (4∙46-5∙94) | 4∙16** | (3∙55 to 4∙87) | 4∙06** | (3∙41-4∙84) | 5∙43** | (4∙36 to 6∙76) | 5∙45** | (4∙36-6∙80) |
|  | **COVID-19** | 1∙17 | (1∙00 to 1∙37) | 1∙16 | (1∙00-1∙35) | 1∙09 | (0∙91 to 1∙31) | 1∙11 | (0∙94-1∙30) | 1∙28 | (0∙96 to 1∙71) | 1∙28 | (0∙96-1∙71) |
|  | **Death and Dying** | 1∙54** | (1∙30 to 1∙83) | 1∙56** | (1∙32-1∙84) | 1∙35** | (1∙11 to 1∙64) | 1∙40** | (1∙16-1∙69) | 1∙37* | (1∙04 to 1∙79) | 1∙33* | (1∙02-1∙74) |
|  | **Resources** | 2∙67** | (2∙20 to 3∙23) | 2∙75** | (2∙24-3∙37) | 2∙37** | (1∙83 to 3∙08) | 2∙34** | (1∙76-3∙13) | 2∙08** | (1∙61 to 2∙68) | 2∙11** | (1∙63-2∙72) |
|  | **Staffing** | 3∙23** | (2∙55 to 4∙10) | 3∙40** | (2∙69-4∙29) | 2∙63** | (1∙92 to 3∙61) | 2∙74** | (1∙97-3∙80) | 3∙48** | (2∙52 to 4∙81) | 3∙35** | (2∙41-4∙66) |

File 2: Odds ratios between the subscales of the practice environment and nurse and patient outcomes by public and private hospital and overall (Unadjusted and Adjusted Odds Ratios)

| **Outcomes** | **Effect** | **NATIONAL** | | | | | **PUBLIC** | | | | **PRIVATE** | | | |
| --- | --- | --- | --- | --- | --- | --- | --- | --- | --- | --- | --- | --- | --- | --- |
|  |  | ***UNADJUSTED*** | | | ***ADJUSTED*** | | ***UNADJUSTED*** | | ***ADJUSTED*** | | ***UNADJUSTED*** | | ***ADJUSTED*** | |
|  |  | **OR** | | **[95% CI]** | **OR** | **[95% CI]** | **OR** | **[95% CI]** | **OR** | **[95% CI]** | **OR** | **[95% CI]** | **OR** | **[95% CI]** |
| **Job dissatisfaction** | PES-NWI:Foundations of Quality of Care | 3∙48** | | (2∙96 to 4∙10) | 3∙39** | (2∙87-4∙01) | 2∙56** | (2∙14 to 3∙05) | 2∙41** | (2∙02-2∙87) | 4∙59** | (3∙47 to 6∙07) | 4∙61** | (3∙49-6∙11) |
|  | PES-NWI:Nurse participation in hospital affairs | 4∙67** | | (4∙08 to 5∙36) | 4∙6** | (3∙99-5∙31) | 3∙47** | (2∙91 to 4∙14) | 3∙26** | (2∙67-3∙98) | 4∙71** | (3∙83 to 5∙79) | 4∙75** | (3∙85-5∙86) |
|  | PES-NWI: Nurse manager, leadership and support  of nurses | 4∙71** | | (3∙97 to 5∙58) | 4∙58** | (3∙85-5∙45) | 3∙15** | (2∙57 to 3∙86) | 2∙91** | (2∙33-3∙63) | 6∙46** | (5∙12 to 8∙14) | 6∙47** | (5∙15-8∙13) |
|  | PES-NWI: Staffing and resource adequacy | 3∙46** | | (2∙84 to 4∙20) | 3∙42** | (2∙78-4∙21) | 2∙81** | (2∙18 to 3∙61) | 2∙56** | (1∙94-3∙36) | 3∙74** | (2∙82 to 4∙95) | 3∙89** | (2∙92-5∙19) |
|  | PES-NWI: Collegial relationships | 2∙68** | | (2∙33 to 3∙08) | 2∙64** | (2∙28-3∙06) | 2∙41** | (2∙04 to 2∙83) | 2∙30** | (1∙91-2∙77) | 3∙50** | (2∙76 to 4∙43) | 3∙51** | (2∙78-4∙44) |
|  | | | | | | | | | | | | | | |
| **Intention to leave** | PES-NWI: Foundations of Quality of Care | | 2∙17** | (1∙77 to 2∙66) | 2∙21** | (1∙81-2∙71) | 1∙73** | (1∙36 to 2∙21) | 1∙76** | (1∙34-2∙30) | 3∙06** | (2∙34 to 4∙01) | 3∙08** | (2∙36-4∙01) |
|  | PES-NWI: Nurse participation in hospital affairs | | 2∙48** | (2∙04 to 3∙02) | 2∙57** | (2∙12-3∙13) | 1∙73** | (1∙32 to 2∙28) | 1∙79** | (1∙34-2∙39) | 3∙69** | (2∙85 to 4∙79) | 3∙79** | (2∙89-4∙98) |
|  | PES-NWI: Nurse manager, leadership and support of nurses | | 2∙81** | (2∙33 to 3∙38) | 2∙87** | (2∙38-3∙47) | 2∙12** | (1∙67 to 2∙70) | 2∙18** | (1∙69-2∙82) | 4∙01** | (3∙09 to 5∙22) | 3∙99** | (3∙05-5∙21) |
|  | PES-NWI: Staffing and resource adequacy | | 2∙04** | (1∙64 to 2∙54) | 2∙05** | (1∙65-2∙55) | 1∙61* | (1∙18 to 2∙19) | 1∙54* | (1∙14-2∙09) | 2∙54** | (1∙88 to 3∙45) | 2∙69** | (1∙94-3∙73) |
|  | PES-NWI: Collegial relationships | | 2∙01** | (1∙69 to 2∙41) | 2∙02** | (1∙68-2∙42) | 1∙54** | (1∙22 to 1∙94) | 1∙52** | (1∙81-1∙96) | 2∙86** | (2∙27 to 3∙61) | 2∙90** | (2∙32-3∙63) |
|  | | | | | | | | | | | | | | |
|  | | | | | | | | | | | | | | |
| **Burnout** | PES-NWI: Foundations of Quality of Care | | 3∙53** | (2∙99 to 4∙16) | 3∙66** | (3∙10-4∙31) | 2∙87** | (2∙37 to 3∙47) | 2∙95** | (2∙43-3∙59) | 4∙08** | (3∙14 to 5∙30) | 4∙24** | (3∙30-5∙43) |
|  | PES-NWI: Nurse participation in hospital affairs | | 4∙05** | (3∙49 to 4∙69) | 4∙11** | (3∙57-4∙74) | 3∙30** | (2∙62 to 4∙16) | 3∙30** | (2∙66-4∙09) | 4∙20** | (3∙50 to 5∙04) | 4∙33** | (3∙59-5∙22) |
|  | PES-NWI: Nurse manager, leadership and support of nurses | | 3∙88** | (3∙34 to 4∙52) | 4∙04** | (3∙46-4∙71) | 3∙37** | (2∙66 to 4∙27) | 3∙50** | (2∙73-4∙47) | 3∙84** | (3∙14 to 4∙69) | 4∙02** | (3∙28-4∙94) |
|  | PES-NWI: Staffing and resource adequacy | | 3∙94** | (3∙30 to 4∙71) | 4∙17** | (3∙50-4∙97) | 3∙00** | (2∙38 to 3∙77) | 3∙17** | (2∙53-3∙98) | 4∙79** | (3∙89 to 5∙89) | 4∙97** | (4∙00-6∙16) |
|  | PES-NWI: Collegial relationships | | 3∙16** | (2∙77 to 3∙61) | 3∙18** | (2∙78-3∙65) | 3∙10** | (2∙58 to 3∙73) | 3∙10** | (2∙56-3∙75) | 3∙35** | (2∙72 to 4∙13) | 3∙36** | (2∙71-4∙15) |
|  | | | | | | | | | | | | | | |
| **Poor mental health** | PES-NWI: Foundations of Quality of Care | | 2∙37** | (1∙98 to 2∙84) | 2∙36** | (1∙95-2∙85) | 2∙06** | (1∙64 to 2∙60) | 2∙02** | (1∙57-2∙60) | 2∙13** | (1∙58 to 2∙86) | 2∙17** | (1∙60-2∙96) |
|  | PES-NWI: Nurse participation in hospital affairs | | 2∙52** | (2∙19 to 2∙90) | 2∙48** | (2∙15-2∙86) | 1∙96** | (1∙55 to 2∙47) | 1∙90** | (1∙49-2∙44) | 2∙28** | (1∙87 to 2∙79) | 2∙33** | (1∙90-2∙85) |
|  | PES-NWI: Nurse manager, leadership and support of nurses | | 2∙58** | (2∙19 to 3∙04) | 2∙60** | (2∙20-3∙08) | 2∙30** | (1∙83 to 2∙90) | 2∙31** | (1∙81-2∙95) | 2∙15** | (1∙72 to 2∙68) | 2∙25** | (1∙80-2∙81) |
|  | PES-NWI: Staffing and resource adequacy | | 2∙56** | (2∙17 to 3∙03) | 2∙51** | (2∙12-2∙98) | 1∙83** | (1∙47 to 2∙29) | 1∙82** | (1∙44-2∙29) | 2∙77** | (2∙26 to 3∙40) | 2∙73** | (2∙21-3∙36) |
|  | PES-NWI: Collegial relationships | | 2∙23** | (1∙91 to 2∙61) | 2∙25** | (1∙92-2∙64) | 2∙44** | (1∙86 to 3∙21) | 2∙48** | (1∙87-3∙28) | 2∙07** | (1∙74 to 2∙46) | 2∙06** | (1∙73-2∙46) |
|  | | | | | | | | | | | | | | |
| **Poor physical health** | PES-NWI: Foundations of Quality of Care | | 2∙40** | (1∙76 to 3∙28) | 1,65 | (0∙58-4∙70) | 1∙91* | (1∙18 to 3∙09) | 1,06 | (0∙33-3∙47) | 2∙05** | (1∙35 to 3∙11) | 1,53 | (0∙26-9∙12) |
|  | PES-NWI: Nurse participation in hospital affairs | | 3∙31** | (2∙61 to 4∙19) | 4∙29** | (2∙07-8∙88) | 2∙91** | (1∙79 to 4∙73) | 3∙10* | (1∙21-7∙94) | 2∙39** | (1∙84 to 3∙11) | 2,84 | (0∙86-9∙35) |
|  | PES-NWI: Nurse manager, leadership and support of nurses | | 2∙86** | (2∙17 to 3∙75) | 3∙73** | (1∙70-8∙17) | 2∙90** | (1∙67 to 5∙03) | 2∙92* | (1∙14-7∙47) | 1∙97** | (1∙49 to 2∙60) | 2,57 | (0∙75-8∙82) |
|  | PES-NWI: Staffing and resource adequacy | | 3∙23** | (2∙65 to 3∙92) | 2∙85** | (1∙51-5∙38) | 2∙28** | (1∙60 to 3∙23) | 2∙08* | (1∙00-4∙30) | 3∙11** | (2∙46 to 3∙92) | 1,66 | (0∙56-4∙95) |
|  | PES-NWI: Collegial relationships | | 2∙46** | (1∙90 to 3∙20) | 2∙85* | (1∙24-6∙54) | 3∙41** | (2∙14 to 5∙45) | 2,69 | (0∙85-8∙51) | 2∙09** | (1∙56 to 2∙79) | 2,64 | (0∙86-8∙04) |
|  | | | | | | | | | | | | | | |
| **Not recommend hospital to  family and friends** | PES-NWI: Foundations of Quality of Care | | 4∙43** | (3∙66 to 5∙36) | 4∙47** | (3∙68-5∙43) | 3∙15** | (2∙64 to 3∙77) | 3∙14** | (2∙62-3∙77) | 7∙05** | (4∙99 to 9∙96) | 7∙02** | (4∙97-9∙92) |
|  | PES-NWI: Nurse participation in hospital affairs | | 4∙95** | (4∙08 to 6∙01) | 5∙02** | (4∙15-6∙09) | 3∙29** | (2∙82 to 3∙84) | 3∙30** | (2∙86-3∙81) | 6∙47** | (4∙58 to 9∙12) | 6∙49** | (4∙59-9∙17) |
|  | PES-NWI: Nurse manager, leadership and support of nurses | | 5∙13** | (4∙29 to 6∙13) | 5∙12** | (4∙24-6∙18) | 3∙75** | (3∙19 to 4∙42) | 3∙70** | (3∙05-4∙48) | 6∙42** | (4∙51 to 9∙13) | 6∙41** | (4∙52-9∙10) |
|  | PES-NWI: Staffing and resource adequacy | | 3∙40** | (2∙78 to 4∙15) | 3∙51** | (2∙86-4∙31) | 2∙56** | (2∙07 to 3∙15) | 2∙52** | (2∙05-3∙11) | 4∙40** | (3∙01 to 6∙41) | 4∙54** | (3∙08-6∙70) |
|  | PES-NWI: Collegial relationships | | 2∙95** | (2∙45 to 3∙56) | 2∙93** | (2∙43-3∙55) | 2∙55** | (1∙95 to 3∙32) | 2∙46** | (1∙88-3∙22) | 4∙45** | (3∙20 to 6∙19) | 4∙49** | (3∙22-6∙26) |
|  | | | | | | | | | | | | | | |
|  | | | | | | | | | | | | | | |
| **Not confident patients can  manage care** | PES-NWI: Foundations of Quality of Care | | 4∙26** | (3∙57 to 5∙08) | 3∙83** | (3∙22-4∙57) | 2∙66** | (2∙18 to 3∙26) | 2∙77** | (2∙22-3∙44) | 5∙05** | (3∙88 to 6∙59) | 5∙19** | (3∙95-6∙81) |
|  | PES-NWI: Nurse participation in hospital affairs | | 6∙51** | (5∙79 to 7∙32) | 3∙82** | (3∙31-4∙42) | 3∙01** | (2∙45 to 3∙69) | 3∙10** | (2∙53-3∙81) | 3∙52** | (2∙85 to 4∙35) | 3∙55** | (2∙86-4∙41) |
|  | PES-NWI: Nurse manager, leadership and support of nurses | | 5∙23** | (4∙44 to 6∙15) | 3∙38** | (2∙94-3∙89) | 2∙57** | (2∙14 to 3∙09) | 2∙63** | (2∙20-3∙14) | 3∙42** | (2∙73 to 4∙29) | 3∙61** | (2∙84-4∙56) |
|  | PES-NWI: Staffing and resource adequacy | | 5∙09** | (4∙36 to 5∙94) | 3∙27** | (2∙74-3∙90) | 2∙25** | (1∙81 to 2∙79) | 2∙21** | (1∙78-2∙75) | 4∙63** | (3∙54 to 6∙05) | 4∙51** | (3∙42-5∙96) |
|  | PES-NWI: Collegial relationships | | 2∙85** | (2∙46 to 3∙30) | 2∙43** | (2∙13-2∙77) | 2∙16** | (1∙82 to 2∙57) | 2∙10** | (1∙74-2∙46) | 3∙17** | 2∙58 to 3∙88) | 3∙14** | (2∙54-3∙87) |
|  | | | | | | | | | | | | | | |
|  | | | | | | | | | | | | | | |
| **Not confident management can resolve patient problems** | PES-NWI: Foundations of Quality of Care | | 4∙88** | (4∙02 to 5∙93) | 4∙22** | (3∙49-5∙10) | 3∙60** | (2∙91 to 4∙45) | 3∙47** | (2∙74-4∙41) | 4∙36** | (3∙16 to 6∙03) | 4∙34** | (3∙11-6∙05) |
|  | PES-NWI: Nurse participation in hospital affairs | | 4∙54** | (3∙75 to 5∙50) | 6∙31** | (5∙60-7∙11) | 5∙76** | (4∙70 to 7∙06) | 5∙19** | (4∙24-6∙36) | 5∙92** | (4∙97 to 7∙04) | 5∙97** | (5∙02-7∙09) |
|  | PES-NWI: Nurse manager, leadership and support of nurses | | 4∙25** | (3∙52 to 5∙14) | 5∙18** | (4∙38-6∙11) | 4∙50** | (3∙48 to 5∙81) | 4∙28** | (3∙28-5∙60) | 5∙01** | (4∙07 to 6∙15) | 5∙00** | (4∙08-6∙14) |
|  | PES-NWI: Staffing and resource adequacy | | 4∙67** | (3∙78 to 5∙76) | 4∙92** | (4∙22-5∙75) | 4∙30** | (3∙56 to 5∙20) | 3∙82** | (3∙16-4∙62) | 5∙40** | (4∙05 to 7∙20) | 5∙52** | (4∙21-7∙24) |
|  | PES-NWI: Collegial relationships | | 3∙14** | (2∙62 to 3∙76) | 2∙83** | (2∙43-3∙30) | 3∙01** | (2∙42 to 3∙74) | 2∙97** | (2∙35-3∙76) | 2∙81** | (2∙29 to 3∙45) | 2∙78** | (2∙25-3∙43) |
|  | | | | | | | | | | | | | | |
| **Poor/Fair ward quality** | PES-NWI: Foundations of Quality of Care | | 3∙67** | (3∙09 to 4∙36) | 4∙93** | (4∙08-5∙97) | 3∙90** | (3∙05 to 4∙98) | 3∙98** | (3∙13-5∙05) | 5∙94** | (4∙42 to 7∙98) | 5∙80** | (4∙29-7∙84) |
|  | PES-NWI: Nurse participation in hospital affairs | | 3∙74** | (3∙22 to 4∙33) | 4∙60** | (3∙77-5∙60) | 3∙58** | (2∙83 to 4∙52) | 3∙63** | (2∙84-4∙64) | 4∙60** | (3∙54 to 5∙96) | 4∙57** | (3∙52-5∙93) |
|  | PES-NWI: Nurse manager, leadership and support of nurses | | 3∙28** | (2∙83 to 3∙79) | 4∙36** | (3∙61-5∙26) | 3∙20** | (2∙58 to 3∙96) | 3∙28** | (2∙63-4∙09) | 5∙07** | (3∙85 to 6∙66) | 5∙10** | (3∙86-6∙71) |
|  | PES-NWI: Staffing and resource adequacy | | 3∙27** | (2∙72 to 3∙93) | 4∙78** | (3∙86-5∙92) | 3∙27** | (2∙61 to 4∙09) | 3∙34** | (2∙64-4∙24) | 7∙24** | (4∙79 to 10∙95) | 7∙22** | (4∙80-10∙85) |
|  | PES-NWI: Collegial relationships | | 2∙47** | (2∙17 to 2∙81) | 3∙16** | (2∙61-3∙83) | 2∙95** | (2∙28 to 3∙81) | 3∙00** | (2∙28-3∙96) | 3∙73** | (2∙81 to 4∙95) | 3∙64** | (2∙75-4∙84) |
|  | | | | | | | | | | | | | | |
| **Poor/Failing safety grade** | PES-NWI: Foundations of Quality of Care | | 4∙21** | (3∙51 to 5∙06) | 4∙33** | (3∙58-5∙24) | 3∙63** | (2∙94 to 4∙49) | 3∙68** | (2∙93-4∙61) | 4∙16** | (3∙17 to 5∙47) | 4∙19** | (3∙19-5∙50) |
|  | PES-NWI: Nurse participation in hospital affairs | | 4∙35** | (3∙81 to 4∙95) | 4∙35** | (3∙81-4∙96) | 3∙54** | (3∙04 to 4∙12) | 3∙54** | (3∙04-4∙12) | 3∙98** | (3∙23 to 4∙91) | 3∙98** | (3∙23-4∙91) |
|  | PES-NWI: Nurse manager, leadership and support of nurses | | 3∙48** | (2∙99 to 4∙04) | 3∙47** | (2∙97-4∙05) | 2∙86** | (2∙40 to 3∙41) | 2∙73** | (2∙28-3∙28) | 3∙31** | (2∙73 to 4∙02) | 3∙30** | (2∙72-4∙01) |
|  | PES-NWI: Staffing and resource adequacy | | 4∙28** | (3∙62 to 5∙05) | 4∙39** | (3∙71-5∙19) | 3∙27** | (2∙71 to 3∙94) | 3∙14** | (2∙56-3∙86) | 5∙37** | (3∙87 to 7∙36) | 5∙41** | (3∙96-7∙39) |
|  | PES-NWI: Collegial relationships | | 2∙62** | (2∙27 to 3∙02) | 2∙65** | (2∙29-3∙07) | 2∙46** | (2∙05 to 2∙95) | 2∙46** | (2∙04-2∙97) | 3∙10** | (2∙43 to 3∙96) | 3∙08** | (2∙40-3∙95) |
